# Video ocular counter roll: A bedside test of otolith‐ocular function

**DOI:** 10.1002/acn3.51921

**Published:** 2023-10-13

**Authors:** Yuchen Yang, Jing Tian, Jorge Otero‐Millan, Amir Kheradmand

**Affiliations:** ^1^ Department of Neurology The Johns Hopkins University School of Medicine Baltimore Maryland USA; ^2^ Herbert Wertheim School of Optometry & Vision Science University of California Berkeley California USA; ^3^ Department of Otolaryngology‐Head and Neck Surgery The Johns Hopkins University School of Medicine Baltimore Maryland USA; ^4^ Department Neuroscience The Johns Hopkins University School of Medicine Baltimore Maryland USA

## Abstract

Here we review the clinical value of a video‐oculography test for clinical evaluation of vestibular otolith function. This test is known as the video ocular counter roll (vOCR) and is based on measurement of torsional vestibulo‐ocular reflex with a lateral head tilt. The vOCR test consists of a simple maneuver during which the head and torso are tilted en bloc by the examiner. The pattern of vOCR deficit among patients highlights its clinical value in identifying the stage of vestibular loss and recovery. The quick application of vOCR allows examination of otolith‐ocular function and assessment of vestibular recovery at the bedside.

Portable video‐oculography (VOG) has been successfully integrated into the bedside evaluation of vestibular functions. As part of this integration, the video head impulse testing (vHIT) is now widely used to examine individual semicircular canals.^1^ Lacking, however, has been a comparable VOG test for clinical evaluation of otolith function. The otolith organs play a key role within the vestibular system and contribute to a wide range of functions including the vestibulo‐ocular reflex (VOR), perception of self‐motion, spatial orientation, and postural control.

Functioning as sensors that can detect linear acceleration, the otolith organs are stimulated by the force of gravity when the head is tilted laterally. This results in a torsional VOR known as the ocular counter roll (OCR), during which the eyes roll in the opposite direction of the head tilt.^2^ During the head movement, the combination of inputs from the otolith organs and semicircular canals results in a torsional nystagmus with the slow phase in the opposite direction of the head tilt. In a stationary head tilt position, however, the static OCR is primarily driven by the inputs from the otolith organs (utricle). This static response has a normal gain (eye position/head position) of about 0.15 (e.g., 4.5^o^ OCR during 30^o^ head tilt).^3^, ^4^

Building on the principles of otolith‐ocular function and OCR physiology, we have developed a VOG test for clinical evaluation of otolith‐ocular function.^3^, ^4^ This test is known as the video OCR (vOCR) and consists of a simple bedside maneuver while the patient is fixing on a visual target in front of them. The head and torso are tilted en bloc 30^o^ by the examiner, which can be comfortably sustained while the patient is sitting on a chair (usually for 30s) (Fig. 1A). Previous studies have shown that the en bloc tilt is more directly associated with otolith function compared with a head tilt on the torso.^5^, ^6^ This is because the vOCR response during the en bloc tilt of the head cannot be confounded by the neck inputs. The vOCR measurement involves tracking the iris to quantify changes in the torsional eye position during the tilt maneuver.^4^, ^7^ A polar transformation is applied to the iris pattern (Fig. 1A) and the image is optimized to enhance the iris features and mask the parts covered by the eyelids.^7^ The iris pattern is set as the baseline reference in the upright position and a template‐matching method is implemented to compare the iris pattern at any point in time during head tilt with the reference value in the upright position.^7^ The position of the head is also recorded in real time using an accelerometer imbedded in the VOG goggles. This real‐time reading is used by the examiner to monitor the tilt angle and prevent movement of the head in other planes during the tilt maneuver.

**Figure 1:**
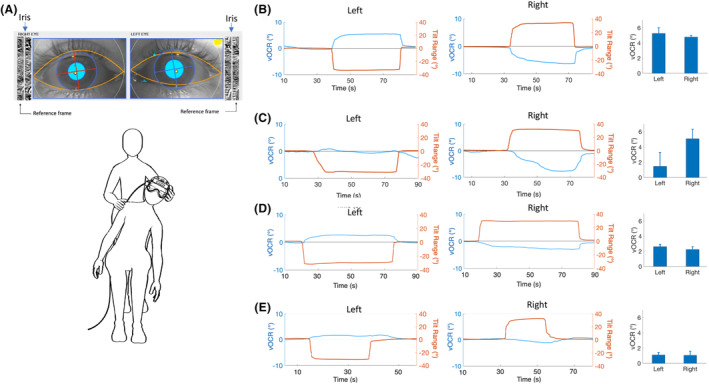
(A) The tilt maneuver in the vOCR test is a passive 30^o^ en bloc tilt of the head and trunk. The vOCR is measured as the torsional eye position (red/blue crosses) by tracking the iris pattern and comparing it to a reference frame in the upright position.^7^ Examples of vOCR traces in (B) a healthy individual, (C) an acute patient (Day 2) with a left‐side vestibular deficit, (D) a chronic patient (>2 years) with a left‐side vestibular loss, and (E) a chronic patient (>2 years) with bilateral vestibular deficit. The vOCR results are shown as the blue trace/axis for the eye position and the orange trace/axis for the head position (negative indicates left and positive indicates right). The mean vOCR values for three tilts on each side are shown in the bar graphs. Informed consent was obtained from all subjects.

The vOCR test is clinically valuable to detect vestibular deficits affecting the otolith organs.^3^, ^4^ The measurements are noninvasive and quick (approximately 5 min to complete), and an automated analysis software can provide instant results. The diagnostic accuracy of vOCR is comparable to the vestibular evoked myogenic potentials (VEMP), a widely used laboratory test of otolith function.^3^ With vOCR values below 4^o^ at 30^o^ head tilt, the accuracy for detecting vestibular deficit is 83%.^4^ The pattern of vOCR deficit varies depending on the time from vestibular injury.^4^ With acute loss of vestibular function (~4 weeks), vOCR is primarily reduced on the affected side, but with chronic vestibular loss, there is a symmetrical reduction on the side of vestibular loss as well as the healthy side (Fig. 1B,C). These findings suggest that vOCR deficit can recover over time after vestibular injury. Such recovery may involve central rebalancing of neural activity within the vestibular system, a process similar to gradual resolution of nystagmus from loss of canal function.^5^, ^6^ In line with this proposed mechanism, the largest vOCR deficit is observed in patients with bilateral vestibular loss (Fig. 1D).^3^

The pattern of vOCR deficit among patients highlights its clinical value in identifying the stage of vestibular loss (Fig. 1).^4^ In the acute stage of vestibular loss, vOCR and vHIT are impaired on the lesion side, allowing both tests to identify the side of vestibular deficit. In the chronic stage, however, while vHIT is impaired on the lesion side, vOCR is symmetrically reduced on both sides.^3^, ^4^ Considering these differences, vHIT and vOCR can be complementary if combined into a single VOG battery (Fig. 2). The vOCR test can detect the stage of vestibular loss in addition to the assessment of otolith function, while vHIT can identify the side of vestibular loss in addition to the assessment of canal function.

**Figure 2:**
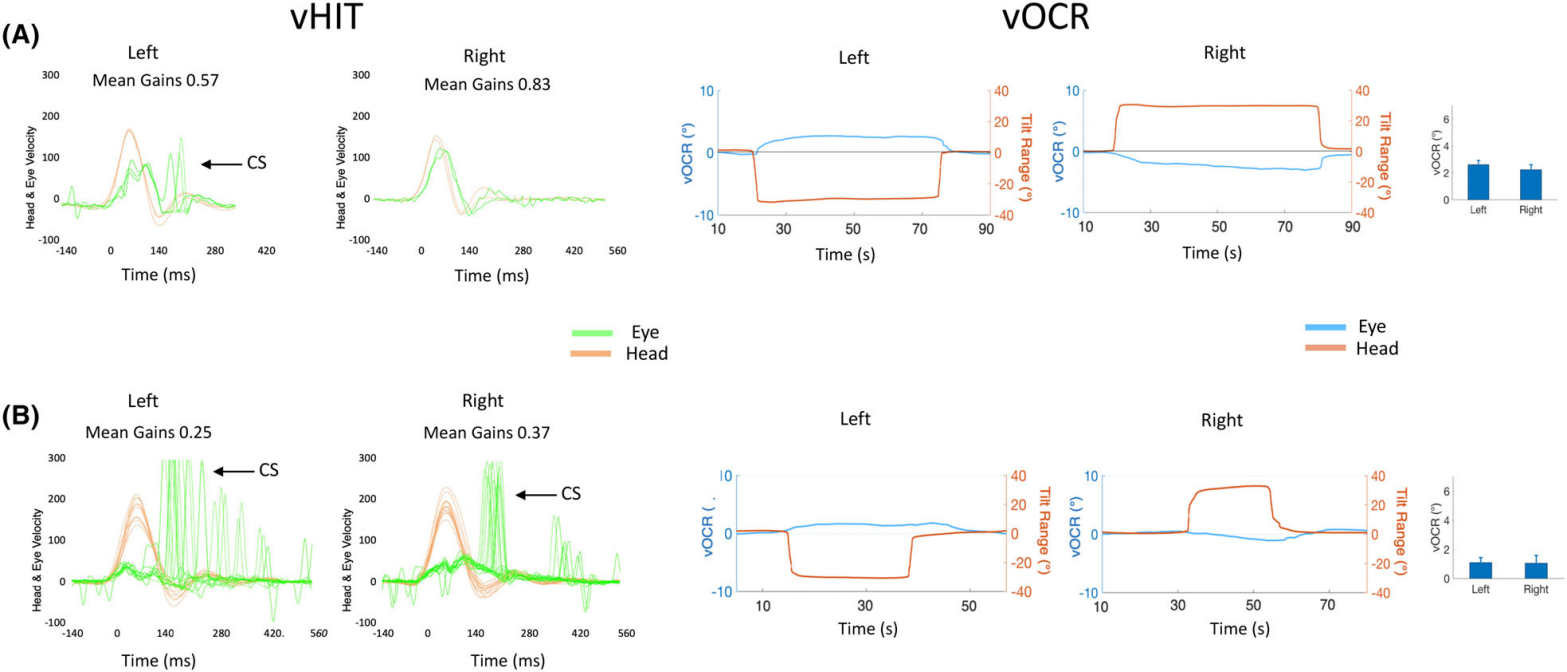
vHIT and vOCR results in two patients with (A) chronic unilateral (left side) vestibular loss and (B) bilateral vestibular loss. The vOCR traces show symmetrically reduced eye position (blue) with head tilt (orange) in the chronic stage (normal values >4^o^ / negative values indicate left and positive values indicate right). Bar graphs show the mean vOCR values for three tilts on each side. The vHIT traces show superimposed velocities of the head (orange) and eye (green) with lateral head impulses. The vHIT gain (eye velocity/head velocity) is reduced on the left side in the unilateral patient and reduced on both sides in the bilateral patient (normal gain >0.8). There are also compensatory catch‐up saccades (CS) on the side of vestibular loss in both patients. Informed consent was obtained from all subjects.

The vOCR test can also be performed with the head tilted on the torso. The effect of neck in this maneuver can enhance vOCR responses in patients with vestibular deficits (Fig. 3). This finding has clinical implications for assessing the compensatory role of neck inputs in the process of recovery following vestibular loss.^5^, ^6^

**Figure 3:**
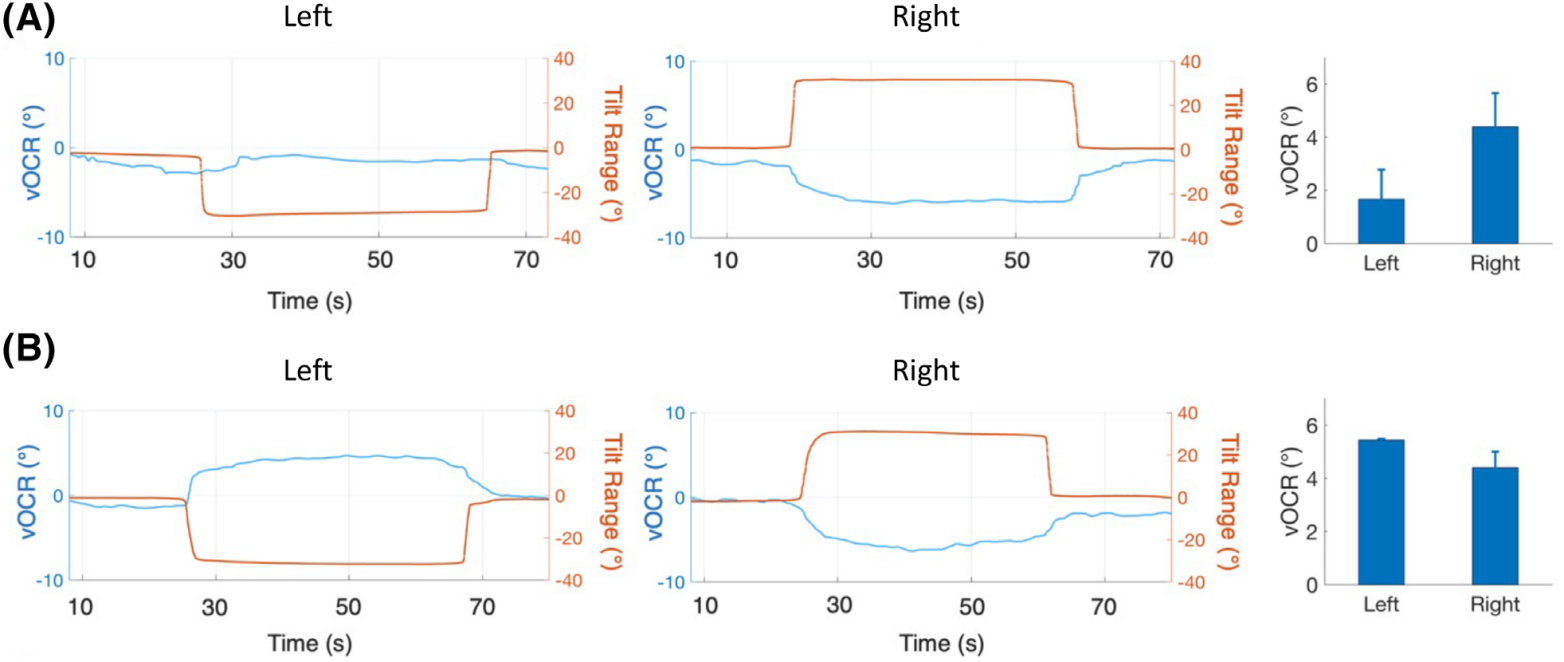
vOCR results from a patient with subacute vestibular loss (7 weeks) with the head and body tilted en bloc (A) and with the head tilted on the body (B). When the head is tilted on the body, the neck effect can enhance the vOCR response on the side of vestibular loss (left side). The vOCR trace is shown as the blue trace/axis for the eye position and the orange trace/axis for the head position (negative indicates left and positive indicates right). The mean vOCR values for three tilts on each side are shown in the bar graphs. Informed consent was obtained from all subjects.

Altogether, the quick application of vOCR allows examination of otolith‐ocular function and assessment of vestibular recovery at the bedside. Future studies should examine whether such recovery corresponds with improvement of daily function and vestibular symptoms in patients. When combined with vHIT, vOCR can identify the stage of vestibular loss and vHIT can detect the side of vestibular loss.

## Author Contributions

Y.Y., A.K., J.T. and J.O.M. contributed to the drafting and revision of the manuscript for content. All authors provided final approval of the manuscript for submission. Y.Y. collected the data and Y.Y., A.K., J.T. and J.O.M. analyzed and/or interpreted the data.

## Funding Information

This work was supported by grants from the National Institute on Deafness and Other Communication Disorders (NIDCD; R01DC018815) and the Leon Levy foundation.

## Conflict of Interest

The authors have no conflict of interest to declare.
